# Risk Management of Hazardous Materials in Manufacturing Processes: Links and Transitional Spaces between Occupational Accidents and Major Accidents

**DOI:** 10.3390/ma11101915

**Published:** 2018-10-09

**Authors:** Francisco Brocal, Cristina González, Genserik Reniers, Valerio Cozzani, Miguel A. Sebastián

**Affiliations:** 1 Department of Physics, Systems Engineering and Signal Theory, Escuela Politécnica Superior, Universidad de Alicante, Campus de Sant Vicent del Raspeig s/n, 03690 Sant Vicent del Raspeig, Alicante, Spain; 2 Manufacturing and Construction Engineering Department, ETS de Ingenieros Industriales, Universidad Nacional de Educación a Distancia, Calle Juan del Rosal, 12, 28040 Madrid, Spain; cggaya@ind.uned.es (C.G.); msebastian@ind.uned.es (M.A.S.); 3 Faculty of Technology, Policy and Management, Safety and Security Science Group (S3G), TU Delft, 2628 BX Delft, The Netherlands; G.L.L.M.E.Reniers@tudelft.nl; 4 Faculty of Applied Economics, Antwerp Research Group on Safety and Security (ARGoSS), University Antwerp, 2000 Antwerp, Belgium; 5 Department of Civil, Chemical, Environmental, and Materials Engineering, Università di Bologna, Via Terracini, 28, 40131 Bologna, Italy; valerio.cozzani@unibo.it

**Keywords:** risk assessment, dangerous substance, Directive 89/391/EEC, Directive 2012/18/EU, ISO 45001:2018 standard, emerging risk, major accident, manufacturing, occupational accident, risk management

## Abstract

Manufacturing processes involving chemical agents are evolving at great speed. In this context, managing chemical risk is especially important towards preventing both occupational accidents and major accidents. Directive 89/391/EEC and Directive 2012/18/EU, respectively, are enforced in the European Union (EU) to this end. These directives may be further complemented by the recent ISO 45001:2018 standard regarding occupational health and safety management systems. These three management systems are closely related. However, scientific literature tackles the researching of these accidents independently. Thus, the main objective of this work is to identify and analyse the links and transitional spaces between the risk management of both types of accident. Among the results obtained, three transitional spaces can be pointed out which result from the intersection of the three systems mentioned. Similarly, the intersection of these spaces gives shape to a specific transitional space defined by the individual directives linked to Directive 89/391/EEC. These results are limited from a regulatory and technical perspective. Thus, the results are a starting point towards developing models that integrate the management systems studied.

## 1. Introduction

In the past few years, manufacturing processes involving chemical substances have evolved to a great extent, from the new technologies applied to these processes to the new products and materials produced and their regulatory framework. The chemical industry is at the heart of the European Union (EU) manufacturing industry, representing approximately 7% of EU industrial production and a 1.1% share of EU GDP. It supplies two-thirds of its production to other sectors within the manufacturing industry [1].

In regard to the regulatory framework in the EU, two relatively-recent regulations stand out at first glance. Regulation (EC) No 1907/2006 on Registration, Evaluation, Authorisation and Restriction of Chemicals (REACH Regulation), aims to protect human health and the environment by ensuring greater safety in the production and use of chemical substances. The REACH Regulation, which entered into force in 2007, applies to all chemical substances and thus has an impact on many businesses [2]. The classification and labelling of hazardous chemicals is governed by Regulation (EC) No 1272/2008 on classification, labelling and packaging of substances and mixtures (CLP Regulation). The CLP Regulation entered into force in 2009 and it enables identification of dangerous substances by means of classification and labelling, and informing users about their hazards through standard symbols and phrases [3].

**Figure 1:**
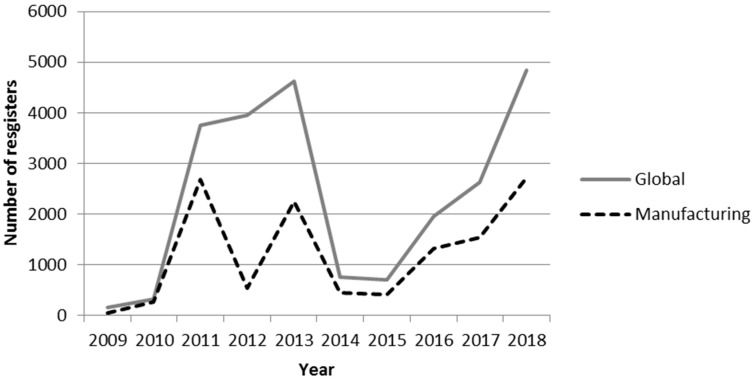
Evolution in the number of chemical substances registered in the European Economic Area under the Registration, Evaluation, Authorisation and Restriction of Chemicals (REACH) regulation. January 2009–August 2018 [5].

In 2018, approximately 145,000 substances were classified according to the CLP Regulation [4]. Also in 2018, more than 21,000 substances were registered in the European Economic Area under the REACH Regulation, of which more than 12,000 are used in manufacturing processes. Figure 1 shows the evolution in the number of chemical substances registered in the European Economic Area under REACH [5] starting in January 2009, when records first exist, until August 2018. To this end, taking into account the uses and exposure to these substances, a distinction is made between their global life cycle and the life cycle applied to manufacturing.

This context of change generates both opportunities and challenges in many fields of knowledge. Among these fields, risk management is particularly important, both from a systemic point of view and from more specific perspectives. Among these specific perspectives, chemical risk management should be noted in order to prevent both occupational accidents and major accidents in manufacturing environments where hazardous materials are used. For this, two separate and solid legislative frameworks exist in the EU.

In the case of the risk management of occupational accidents, Directive 89/391/EEC on the introduction of measures to encourage improvements in occupational safety and health (OSH) should be pointed out [6]. This directive has been developed through a broad set of specific directives, with Directive 98/24/EC on OSH in the field of chemical agents [7] standing out in the context of hazardous materials. 

In the case of the risk management of major accidents and hazards involving dangerous substances, Directive 2012/18/EU is applicable [8]. This directive states that its provisions should be applied without prejudice to the provisions of Union law relating to OSH and the working environment, and, in particular, without prejudice to Council Directive 89/391/EEC [6].

Hence, these risk management frameworks are closely related [9] and, as a result, are open to being studied from an integrative point of view. However, despite the importance of this relationship, scientific literature addresses the study of risk management of occupational accidents and major accidents involving dangerous materials in an independent and practically excluding manner.

In this regard, using the ScienceDirect database [10], the search for the keyword ‘Directive 2012/18/EU’ anywhere in an article, returns 13,584 papers. Similarly, the keyword ‘Directive 89/391/EEC’ returns 473 papers. However, combining both keywords with the AND operator returns three articles, which are: Rasmussen et al. [11], Besserman and Mentzer [12], and Li and Guldenmund [13].

This circumstance defines a closed border between these management systems, making it difficult to develop integrative techniques and methodologies that favour the reduction of risk due to hazardous substances. In addition to the described relationship between directives, the recent opportunity defined by the ISO 45001:2018 standard on OSH management systems [14] should be pointed out.

Thus, the main objective of this study is to identify and analyze the links and transitional spaces between the risk management of occupational accidents and major accidents involving hazardous materials. To achieve this goal, the methodology followed by this study is based on researching and carrying out a comparative analysis of the legal and standardised context described above. To this end, this work is organised as follows: (1) study of the legal context within the framework of the EU; (2) comparative analysis between management systems derived from Directive 89/391/EEC [6] and Directive 2012/18/EU [8]; (3) comparative analysis between management systems derived from the ISO 45001:2018 standard [14] and Directive 2012/18/EU [8]; (4) analysis of transitional spaces between risk management of hazardous materials in manufacturing processes; (5) discussion of results; and (6) conclusions.

## 2. Legal Context in the European Union (EU)

Directive 2012/18/EU (hereinafter, Directive Seveso III) is applicable in regard to the management of major accidents and hazards involving dangerous substances. This directive states that its provisions should be applied without prejudice to the provisions of Union law relating to OSH and the working environment, and, in particular, without prejudice to Council Directive 89/391/EEC. 

The object of Directive 89/391/EEC (hereinafter, Framework Directive) is to introduce measures to encourage improvements in OSH. To this end, it contains general principles concerning the prevention of occupational risks, the protection of safety and health, the elimination of risk and accident factors, the informing, consultation, balanced participation in accordance with national laws and/or practices and training of workers and their representatives, as well as general guidelines for the implementation of said principles.

The Framework Directive serves as basis for more specific directives covering all the risks connected with safety and health in the workplace. Thus, 20 specific directives have been enacted to date since 1989, as listed in Table 1. Thus, Directive Seveso III is applicable to serious accidents and the Framework Directive to occupational risk prevention in a wide sense: that is, considering prevention of occupational accidents, illnesses and other dangers to the safety and health of workers.

Considering that the objectives of this study are linked to major accidents and occupational accidents, both concepts will be defined below. Then, the main relationships that exist between the directives involved in the legal context set out here will be analysed.

### 2.1. Major Accident and Occupational Accident

Directive Seveso III defines ‘major accident’ as an occurrence such as a major emission, fire, or explosion resulting from uncontrolled developments in the course of the operation of any establishment covered by this directive, and leading to serious danger to human health or the environment, immediate or delayed, inside or outside the establishment, and involving one or more dangerous substances.

The Framework Directive and its specific directives do not define occupational accidents. To this end, others sources are required. For example, Eurostat defines an occupational accident (or accident at work) as a discrete occurrence during the course of work which leads to physical or mental harm [34]. The ISO 45001:2018 standard defines ‘incident’ as an occurrence arising out of, or in the course of, work that could or does result in injury and ill health. An incident where injury and ill health occurs is sometimes referred to as an ‘accident’ [14]. 

Comparing the definitions for major accidents and occupational accidents, it could be said that a major accident could also be considered an occupational accident whenever there is harm to workers (injury and ill health). Among the specific directives developed following the Framework Directive, Directive 98/24/EC on chemical agents [7] is the one which, in principle, is more closely linked to Directive Seveso III. Taking this directive into account, it could be said that an occupational accident involving chemical substances is an occurrence arising out of, or in the course of, work that could or does result in injury and ill health for workers. All of this is regardless of the level of severity or seriousness of damage.

In any case, there are other directives besides Directive 98/24/EC on chemical agents [7] that are closely linked to Directive Seveso III. In order to identify these directives, the definition of major accident included in Directive Seveso III will be taken into account, considering to this end the occurrence of a major emission, fire, or explosion.

These events are covered by the industrial safety technologies (IST) studied by Sebastián and Brocal [35], which can be defined as follows: a set of instruments and industrial processes that enable the practical use in analysis, evaluation and control of specific risks being able to be classified into: work equipment; places and workplaces; handling, storage and transport; electricity; fires; and chemicals.

Sebastián and Brocal [35] studied the relationship that exists between IST and specific, applicable directives, the (adapted) results of which are included in Table 2. Considering this result, a preliminary approximation is then offered in regard to the technical–legal relationship that exists between the individual directives and Directive Seveso III.

### 2.2. Activity Involving Chemical Agents

Directive 98/24/EC defines ‘Chemical agent’ [7] as any chemical element or compound, on its own or admixed, as it occurs in the natural state or as produced, used or released, including release as waste, by any work activity, whether or not produced intentionally and whether or not placed on the market. In addition, ‘hazardous chemical agent’ means: (a) any chemical agent which meets the criteria for classification as hazardous within any physical and/or health hazard classes laid down in the CLP Regulation, whether or not that chemical agent is classified under that regulation; (b) any chemical agent which, whilst not meeting the criteria for classification as hazardous in accordance with point (a) may, because of its physicochemical, chemical or toxicological properties and the way it is used or is present in the workplace, present a risk to the safety and health of workers, including any chemical agent that is assigned an occupational exposure limit value under Article 3 of this directive. Directive Seveso III defines ‘dangerous substance’ as a substance or mixture covered by Part 1 or listed in Part 2 of its Annex I, including in the form of a raw material, product, by-product, residue or intermediate.

The main difference observed on comparing the definitions of ‘dangerous substance’ (Seveso III) and ‘hazardous chemical agent’ (Directive 98/24/EC [7]), lies in the former referencing specific substances and amounts (Annex I) while the latter has a much broader and general definition, considering any substance that could give rise to an occupational risk, regardless of whether they meet the classification criteria laid down in the CLP Regulation [3]. 

Thus, Directive 98/24/EC [7] will be applicable to any work with dangerous substances according to Seveso III, given that these dangerous substances will also be hazardous chemical agents.

Additionally, Directive 98/24/EC defines ‘Activity involving chemical agents’ as any work in which chemical agents are used, or are intended to be used, in any process, including production, handling, storage, transport or disposal and treatment, or which result from such work [7]. Directive Seveso III defines ‘presence of dangerous substances’ as the actual or anticipated presence of dangerous substances in the establishment, or of dangerous substances which it is reasonable to foresee may be generated during loss of control of the processes, including storage activities, in any installation within the establishment, in quantities equal to or exceeding the qualifying quantities set out in Part 1 or Part 2 of its Annex I.

The main difference observed in regard to criteria regarding the presence of dangerous substances again lies in the variables collected in Annex I of Directive Seveso III. Thus, Directive 98/24/EC [7] will be applicable to any occupational activity involving dangerous substances included in Seveso III.

Directive 98/24/EC on chemical agents [7] is complemented by Directive 2004/37/EC on carcinogens or mutagens at work [20], as collected in Table 2. The CLP Regulation [3] may be considered the connection point between both directives. Similarly, this regulation is also linked closely to Directive Seveso III.

With the aim of establishing these links, Table 3 collects 7 dangerous substances. These 7 substances have been selected as follows: (a) of the 48 substances collected in Annex I, Part 2 of Seveso III, those with a CAS number have been selected, amounting to 35; (b) for each of these 35 substances with a CAS number, the INFOCARQUIM database [36] has been used to determine those which are carcinogens or mutagens (1A/1B) according to the CLP Regulation; (c) from the 35 substances above, 7 substances have been identified as carcinogens or mutagens; (d) for each of these 7 substances, their H statements have been identified with examples of manufacturing processes, also by using the INFOCARQUIM database; (e) for the 7 substances above, their threshold limit values (VLA, *Valor Límite Ambiental*) have been identified according to the document on the limits of chemical agents for professional exposure in Spain [37].

VLA are reference values for chemical agent concentration in the air and represent the conditions for which it is believed that, based on current knowledge, most workers may be exposed to on a daily basis throughout their work life without suffering adverse effects on their health [37].

Thus, the 7 substances mentioned will be subject to the implementation of Directive Seveso III according to the figure collected in columns 2 and 3. Additionally, these substances fall within the scope of Directive 98/24/EC [7] and Directive 2004/37/EC [6] whenever they are found in workplaces. When these substances may be inhaled by workers, applicable threshold limit values must be considered. For Spain, these values are collected in Table 3. 

Although not contained in Table 3, the applicability of other directives and regulations should be analysed for every manufacturing process that is studied; for instance, the directives collected in Table 1. To this end, one of the essential sources of information is the H statement collected in Table 3.

### 2.3. Workplace

Directive 89/654/EEC [15] lays down minimum requirements for safety and health at the workplace. For the purposes of this directive, ’workplace’ means the place intended to house workstations on the premises of the undertaking and/or establishment and any other place within the area of the undertaking and/or establishment to which the worker has access in the course of his employment.

On the other hand, Directive Seveso III shall apply to establishments. Establishment means the whole location under the control of an operator where dangerous substances are present in one or more installations, including common or related infrastructures or activities; establishments are either lower-tier establishments or upper-tier establishments. Thus, establishments under Seveso III are also workplaces under the definition of Directive 89/654/EEC [15].

### 2.4. Installation

Directive Seveso III defines ‘installation’ as a technical unit within an establishment and whether at or below ground level, in which dangerous substances are produced, used, handled or stored; it includes all the equipment, structures, pipework, machinery, tools, private railway sidings, docks, unloading quays serving the installation, jetties, warehouses or similar structures, floating or otherwise, necessary for the operation of that installation.

Also, Directive 2009/104/EC defines ‘work equipment’ as follows: any machine, apparatus, tool or installation used at work [16]. Installations considered to be work equipment are for example: surface treatment installations, painting installations, installations composed of a combination of machines that work interdependently, etc. [38]. As for general service or protection installations, such as electrical installations, gas or fire protection, annexed to the workplace, that are considered as an integral part thereof, then Directive 89/654/EEC on workplaces is applicable [38].

As a result, the concept of installation, as defined by Directive Seveso III, may be considered to be part of the scope of Directive 2009/104/EC on work equipment [16], as well as Directive 89/654/EEC on the workplace [15]. More specifically, Directive 98/24/EC [7] indicates that work equipment and protective systems provided by the employer for the protection of workers shall comply with the relevant EU provisions on design, manufacture and supply with respect to health and safety. Likewise, the employer shall take measures to provide sufficient control of plant, equipment and machinery or provision of explosion suppression equipment or explosion pressure relief arrangements. 

Furthermore, Directive 98/24/EC [7] specifically points out the need to adopt measures in view of explosions linked to work equipment and installations. Thus, a direct link is established between Directive 99/92/EC [28] where ‘explosive atmosphere’ means a mixture with air, under atmospheric conditions, of flammable substances in the form of gases, vapours, mists or dust in which, after ignition has occurred, combustion spreads to the entire unburned mixture. However, for example this directive shall not apply to the manufacture, handling, use, storage and transport of explosives or chemically unstable substances. 

## 3. Comparative Analysis between Management Systems Derived from the Framework Directive and Directive Seveso III

Directive Seveso III indicates that member states shall require the operator to draw up a document in writing setting out the major-accident prevention policy (MAPP) and to ensure that it is properly implemented. The MAPP shall be implemented by appropriate means, structures and by a safety management system, in accordance with Annex III of this directive, and it will be proportionate to the major-accident hazards, and the complexity of the organization or the activities of the establishment. Table 4 shows the structure of the safety management system according to such Annex III. This Annex III is linked, in turn, to Annex II regarding minimum data and information to be considered in the safety report referred to in Article 10 of Directive Seveso III.

The Framework Directive does not explicitly develop a safety management system. However, it contains general principles concerning the prevention of occupational risks, the protection of safety and health, the elimination of risk and accident factors, the informing, consultation, balanced participation in accordance with national laws and/ or practices and training of workers and their representatives, as well as general guidelines for the implementation of the said principles.

In themselves, the aforementioned general principles form the basis for a management system; in this case, a system to manage the safety and health of workers. To develop this, each member state of the European Union must transpose the Framework Directive into their national legal systems. 

By way of example, Law 31/1995 on occupational risk prevention transposes the Framework Directive into Spanish law [39]. This Law explicitly states that occupational risk prevention must be integrated into an undertaking’s general management system, across all of the activities and across the hierarchy thereof, by means of implementing and putting into practice an occupational risk prevention plan. This occupational risk prevention plan should include the organisational structure, responsibility, roles, practices, procedures, processes and resources necessary to carry out risk prevention activity in the undertaking. The management and implementation instruments that are essential to the risk prevention plan are: the assessment of occupational risk and the planning of preventive action.

Also, Royal Decree 39/1997, which validates the Regulation on Prevention Services, develops those aspects that make it possible to integrate occupational risk prevention management into the undertaking’s activities and across the hierarchical levels thereof, based on a plan that includes work techniques, organisation and conditions [40]. Thus, as shown in Table 4, correspondence may be established between the structure of the Framework Directive and the management system included in Annex III of Directive Seveso III. 

Correspondence may be classified as strong, weak or non-existent. Correspondence is strong when an issue in Annex III has its direct equivalence (major risk management vs. occupational risk management) with one or more articles of the Framework Directive. Correspondence is weak when an issue only has partial equivalence. And, obviously, correspondence is non-existent whet there is no equivalence.

Considering this, there is strong correspondence in regard to the following sections of Annex III: (i) Organization and personnel, (ii) Identification and evaluation of major hazards, (iv) Management of change, and (v) Planning for emergencies.

Correspondence is weak in sections (iii) Operational control and (vi) Monitoring performance. In regard to section (iii), the Framework Directive does not explicitly state: the management and control of risks associated with ageing equipment installed in the establishment and corrosion. In regard to section (vi), the Framework Directive does not explicitly state: the adoption and implementation of procedures for the ongoing assessment of compliance with the objectives set by the operator’s MAPP and safety management system; near misses. There is no (explicit) correspondence in regard to section (vii) regarding Audit and review.

Furthermore, the Framework Directive contains specific guidelines in regard to chemical agents. These guidelines may be understood in terms of the relevance of the risk of such agents on occupational risk overall. Specifically, article 6.2 of the directive states as one of the general principals of preventive activity: replacing the dangerous by the non-dangerous or the less dangerous. Also, article 6.3.a: evaluate the risks to the safety and health of workers, inter alia in the choice of work equipment, the chemical substances or preparations used, and the fitting-out of workplaces. In order to carry out such assessment regarding chemical substances, Directive 98/24/EC [7] and Directive 2004/37/EC [6], on chemical agents and on carcinogens and mutagens at work, respectively, are essential.

Considering that these directives are part of the development of the Framework Directive, they share with it the general principles of risk management. As a result, the minimum provisions for the protection of workers contained in each directive must be managed under the said general management principles. Thus, a correspondence may be observed between the said principles and provisions, from the general to the specific.

Such correspondence makes it possible, in turn, to relate the sections of Annex III of Directive Seveso III with the specific provisions of each directive. This correspondence with the aforementioned directives may be seen in Table 5. The level of detail is lower than that found in Table 4, as in this case, the aim is to offer a global overview of the common thread described, from the general (Table 4) to the specific (Table 5).

Thus, comparing the results of Table 4 and Table 5 offers the following main learnings: (a) section (i) Organization and personnel of Directive Seveso III corresponds closely with the Framework Directive, while this correspondence is weaker with the individual directives analysed, limited mainly to training requirements; (b) section (ii) to (vi) of Directive Seveso III correspond closely with the individual directives. This correspondence is the result of going from the general in the Framework Directive to the specific on issues regarding chemical agents and carcinogens and mutagens pursuant to Directive 98/24/EC [7] and Directive 2004/37/EC [20], respectively; (c) in regard to section (vii) on Audit and review, which has no correspondence with the Framework Directive, reviewing the risk assessment whenever necessary is explicitly considered, such as when working conditions change, new scientific knowledge is achieved on the effects of chemical agents or limit values, etc.

## 4. Comparative Analysis between Management Systems Derived from the ISO 45001:2018 Standard and Directive Seveso III

Implementing an OSH management system conforming to the ISO 45001:2018 standard enables an organization to manage its OSH risks and improve its OSH performance [14]. According to this voluntary standard, an OSH management system can assist an organization to fulfil its legal and other requirements. The implementation and maintenance of an OSH management system, its effectiveness and its ability to achieve its intended outcomes are dependent on a number of key factors which can include the integration of the OSH management system into the organization’s business processes and compliance with its legal and other requirements. 

As indicated by the ISO 45001:2018 standard, its adoption in a given organization, however, will not in itself guarantee prevention of work-related injury and ill health to workers, provision of safe and healthy workplaces and improvement of OSH performance [14]. So, in the context of this work, that is from the perspective of a risk management system, the adoption or implementation of the ISO 45001:2018 standard [14] may be understood as a tool that may assist an organisation in complying with the Framework Directive and Directive Seveso III.

As with the previous section, which carried out a comparative analysis between management systems derived from the Framework Directive and Directive Seveso III, this section contains a similar analysis, using the same criteria, comparing between management systems derived from the ISO 45001:2018 standard [14] and Directive Seveso III.

Hence, as Table 5 also shows, correspondence may be established between the structure of the ISO 45001:2018 standard [14] and the management system included in Annex III of Directive Seveso III. Consequently, the result obtained makes it possible to establish a similar correspondence between the said standard and the Framework Directive, since Directive Seveso III works as a common denominator.

The results from such analysis establish a strong correspondence with all sections from Annex III except with section (iii) Operational control, where aspects related to management and control of the risks associated with ageing equipment installed in the establishment and corrosion are not explicitly stated by the ISO 45001:2018 standard [14]. This is also true in regard to the Framework Directive, Directive 98/24/CE on chemical agents [7] and Directive 2004/37/EC on carcinogens or mutagens [20].

As a result of this correspondence, the ISO 45001:2018 standard [14] is linked closely, not only to Directive Seveso III but also to the Framework Directive and its individual directives, especially Directive 98/24/EC [7] and Directive 2004/37/EC [20], on chemical agents and carcinogens or mutagens at work, respectively.

**Figure 2:**
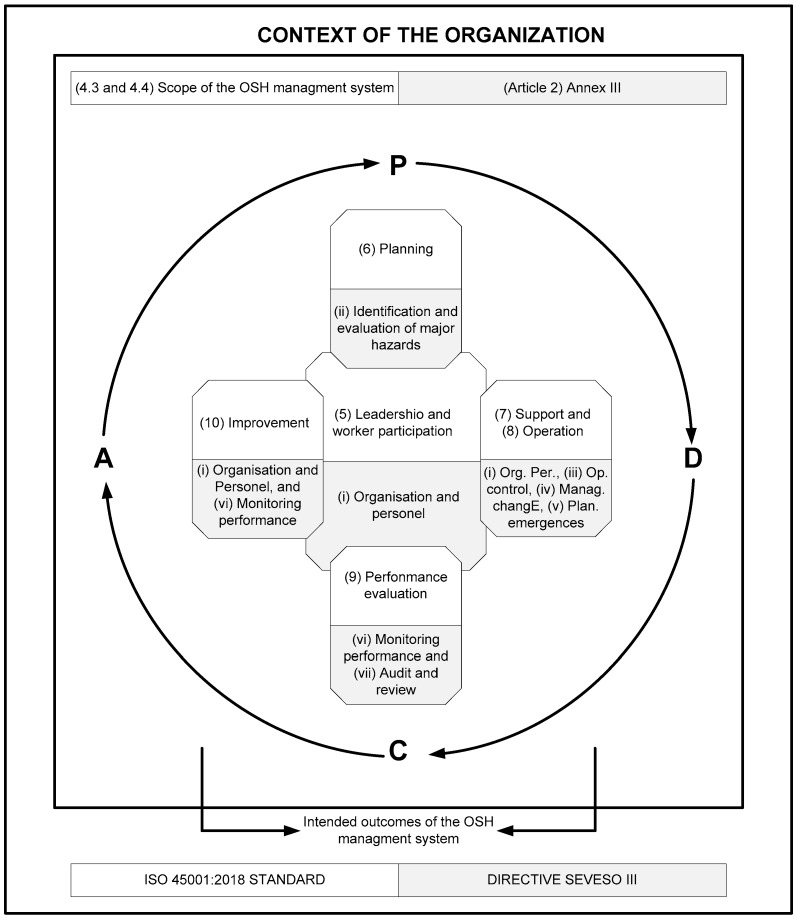
Integration of the Plan-Do-Check-Act (PDCA) framework defined by the ISO 45001:2018 standard [14] with the management system derived from Directive Seveso III [8].

On the other hand, the OSH management system approach defined by the ISO 45001:2018 standard [14] is founded on the concept of Plan-Do-Check-Act (PDCA). This standard indicates that it can be applied to a management system and to each of its individual elements, as follows:

- Plan: determine and assess OSH risks, OSH opportunities and other risks and other opportunities, establish the OSH objectives and processes necessary to deliver results in accordance with the organization’s OSH policy;
- Do: implement the processes as planned;
- Check: monitor and measure activities and processes with regard to the OSH policy and OSH objectives, and report the results;
- Act: take actions to continually improve OSH performance towards achieving the intended outcomes;

The ISO 45001:2018 standard incorporates the PDCA concept into a new framework [14]. This framework can be integrated with the framework defined by Seveso III, considering the results shown in Table 4. The result of such integration is shown in Figure 2.

## 5. Analysis of Transitional Spaces Between the Risk Management of Hazardous Materials in Manufacturing Processes

An analysis is carried out below of the main transitional spaces that arise from the results obtained in the previous sections. Each of these transitional spaces may be understood as the intersection between the correspondences identified between the different sets analysed: that is, the legal context and management systems derived from Directive Seveso III, the Framework Directive and the ISO 45001:2018 standard [14].

### 5.1. Legal Context

**Figure 3:**
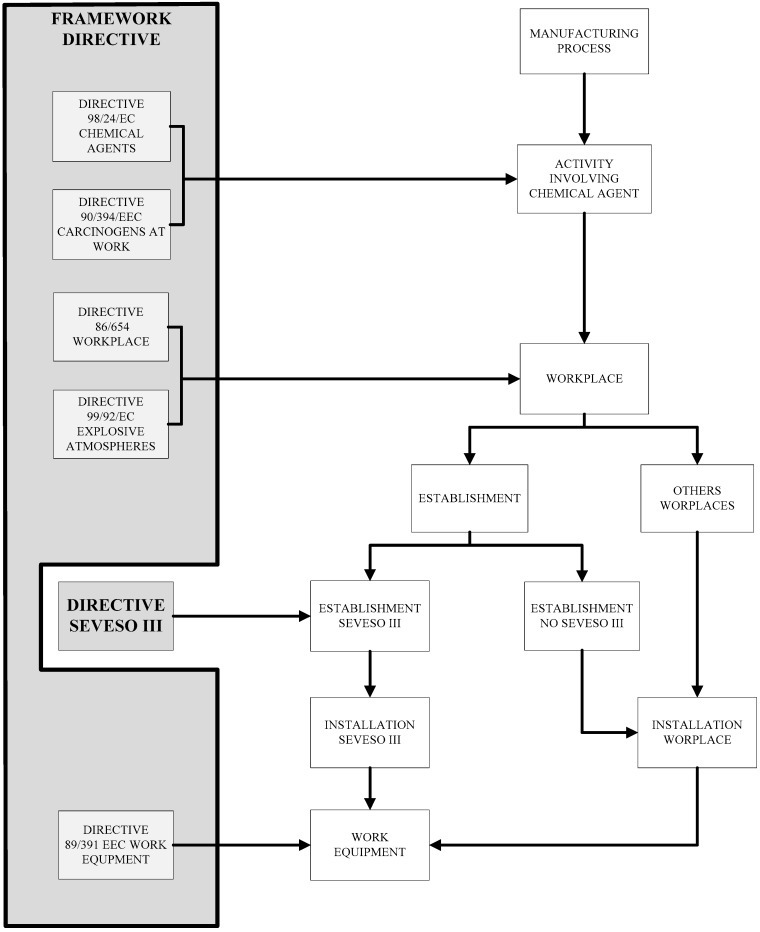
Main links formed between the individual directives (Framework Directive [6]) and Directive Seveso III [8] within the general structure of a manufacturing process.

As a result of the analysis carried out in the legal context section, Figure 3 shows the main correspondence observed between the directives studied; that is, between Directive Seveso III and the individual directives within the meaning of Article 16 (1) of the Framework Directive.

To show these links summarily, the same thread has been followed that configures the structure of the legal context section. To this end, the basic outline of a general manufacturing process has been considered, involving chemical agents (Directive 98/24/EC [7] and Directive 2004/37/EC [20]). Thus, this manufacturing process may take place in one or more workplaces (Directive 89/654/EEC [15]) where, among other chemical risks, there may be the risk of explosion (Directive 99/92/EC [28]). Such workplaces may be considered as establishments with installations in the scope of implementation of Directive Seveso III, other types of establishments (not falling within Directive Seveso III) or other types of workplaces. In all cases, these workplaces will have installations and work equipment (Directive 2009/104/EC [16]) that will configure the corresponding manufacturing process. 

Analysis of directives related to the safety and health of persons other than the individual directives within the meaning of Article 16 (1) of the Framework Directive, are not within the remit of this study. However, it should be pointed out that there may be connections to other regulations, such as the REACH Regulation [2] and CLP Regulation [3], or Directive 2006/42/EC on machinery [41]. These examples may be considered as fundamental regulations in the field of safety and health. 

The main transitional spaces between these links, as shown in Figure 3, are describe below, considering in this regard the intersections between Directive Seveso III and the individual directives within the meaning of Article 16 (1) of the Framework Directive:

- *Activity involving chemical agents:* Directive 98/24/EC on chemical agents [7] will be applicable to any work activity linked to a manufacturing process that involves the use of any of the dangerous substances collected in Directive Seveso III. Additionally, other individual directives may be applicable depending on the characteristics of the manufacturing process in each specific case, as well as the chemical agents used; for instance, Directive 2004/37/EC on carcinogens or mutagens at work [20], as shown for the substances collected in Table 3.
- *Workplaces:* Directive 98/654/EEC on Workplaces [15] is applicable to any work activity linked to a manufacturing process integrated in an establishment, following Directive Seveso III. Additionally, other individual directives may also be applicable depending on the characteristics of the manufacturing process in each specific case, as well as the chemical agents used; for instance, Directive 99/92/EC on explosive atmospheres [28]. This could be the case, for instance, in the case of manufacturing processes that use any of the flammable substances collected in Table 3, such as: ethyleneimine (H225: very flammable liquid and vapours), Ethylene oxide (H220: extremely flammable gas) and propylene oxide (H224: extremely flammable liquid and vapours).
- *Installation:* Directive 2009/104/EC on work equipment [16], as well as Directive 89/654/EEC on workplaces [15], will be applicable to any work activity linked to any manufacturing process that involves one or more installations pursuant to Directive Seveso III. In general, a manufacturing process will be configured by elements that fall within the definition of work equipment and workplace, which may completely or partially configure an installation pursuant to Directive Seveso III.

### 5.2. Management Systems

**Figure 4:**
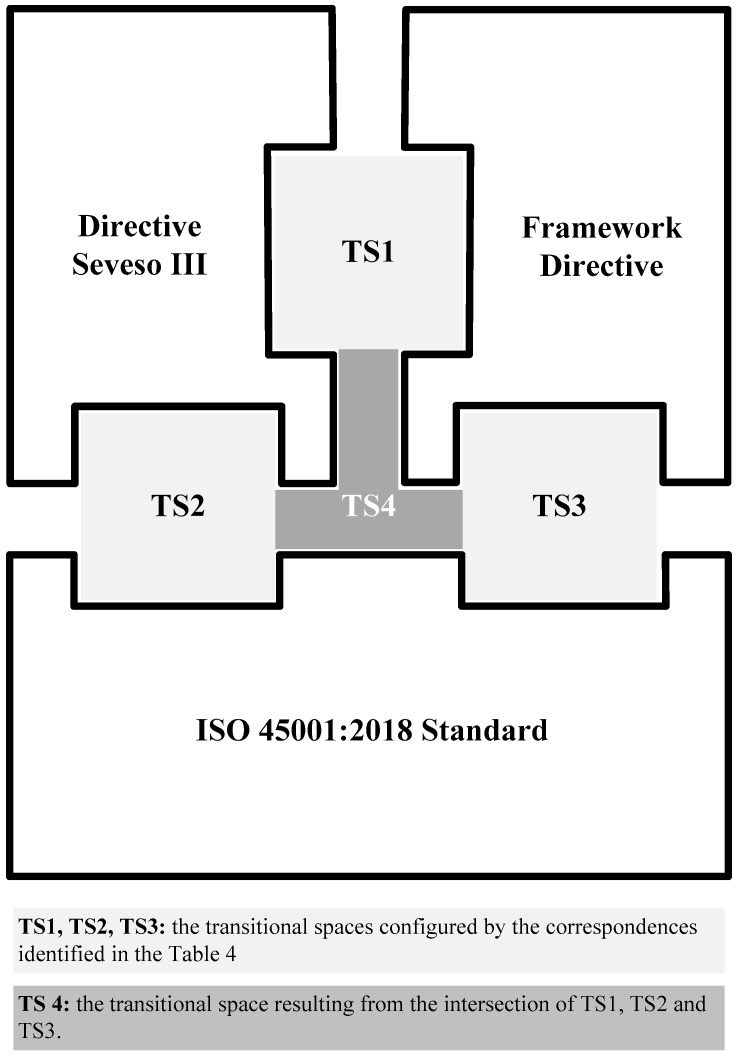
Transitional spaces between the following management systems: Directive Seveso III [8], Framework Directive [7] and ISO 45001:2018 standard [14].

The four transitional spaces (TS) represented conceptually in Figure 4 are derived from the results obtained in the two comparative analyses carried out between risk management systems (the links shown in Table 4 and Table 5): that is, between Directive Seveso III and the Framework Directive, and between Directive Seveso III and the ISO 45001:2018 standard [14].

Transitional space TS 1 is configured by the correspondence that exists between the structure of the Framework Directive and the management systems derived from Directive Seveso III. Similarly, TS 2 is configured by the correspondence that exists between the structure of the ISO 45001:2018 standard and the management system derived from Directive Seveso III. 

Transitional space TS 3 may be considered a ‘natural space’ between the Framework Directive and the ISO 45001:2018 standard [14] since the goal of both systems is to manage safety and health at work. However, studying this transitional space falls outside the aim of this study as such a study would form part of an analysis process regarding the implementation of the ISO 45001:2018 standard [14] by an organisation.

In any case, given that the result of implementing the ISO 45001:2018 standard [14] may be understood as a tool that can help an organisation to meet such legal requirements as complying with the Framework Directive and Directive Seveso III, the three systems may share an intersectional space, giving rise to transitional space TS 4.

Additionally, given that the provisions of Directive 98/24/EC on chemical agents [7] and Directive 2004/37/EC on carcinogens or mutagens at work [20] are in line with the general management guidelines set by the Framework Directive, the correspondence between these directives and Annex III of Directive Seveso III has been studied and the results are collected in Table 5.

The said results may be considered as a specific correspondence transferred from transitional space TS 1 to transitional space TS 4 when the intersection of the three systems takes place. Thus, TS 4 may be considered to include, at least, the provisions of Directive 98/24/EC [7] and Directive 2004/37/EC [20]. In other words, transitional space TS 4 may be considered to be a specific transitional space resulting from the intersection of three general transitional spaces. 

Even if this specific transitional space has been studied in regard to the aforementioned directives, it should be pointed out that other directives, from those considered in Table 1, may also be of interest. 

## 6. Discussion

There are signs of a revitalizing interest in foundational issues in risk assessment and management, which is welcome and necessary for meeting the challenges currently faced by the field of risk. These are related to societal problems and complex technological and emerging risks [42] which can exist in manufacturing processes alongside traditional risks [43]. 

The second European Survey of Enterprises on New and Emerging Risks reveals that dangerous substances (or biological substances) are most prevalent in the European Union in certain sectors such as manufacturing (51.7%) [44]. As a result, new challenges for the management of dangerous substances in the workplace are emerging, for example, in the area of green jobs (bio-energy production, new types of energy storage) and in relation to the use of innovative materials (e.g. nanomaterials) and technologies with currently unknown health risks (such as 3D printing) and substances recognised as endocrine disrupters [45].

In this context, Brocal et al. [46] considered that the relationship between the prevention of occupational accidents and major accidents is especially important. Based on this integrative perspective, Zio [47] points out to the realization that, to manage risk in a systematic and effective way, it is necessary to consider all phases of potential accident scenarios together.

There is a general research focus on dynamic risk assessment and management rather that static or traditional risk assessment [42], which can consider the dynamic evolution of conditions, both internal and external to the system, affecting risk assessment [48]. The effectiveness in the application of a dynamic risk management framework in collecting and considering evidence of emerging risks relies on the continuous development of dynamic techniques for hazard identification and risk assessment, joined with a proper safety culture [49]. 

However, this study does not distinguish between the risk management of both types of risks; that is, between traditional risk and emerging risk. This is due to the general characteristics of the legal and standardised context in which this study has been carried out, towards meeting the main objective set out.

In any case, this objective may be extended in future research in the direction of emerging risk management. In this regard, the CWA 16649:2013 standard on managing emerging technology-related risks may be considered to be a reference management system [50]. Such a direction may be justified, among others, by considering the data in Figure 1, which shows that the number of chemical substances used in manufacturing processes has increased in the past few years. Furthermore, by considering the hypothesis that, in general, such substances are a source of risk, implementing the TICHNER (Technique to Identify and CHaracterize NERs) technique developed by Brocal et al. [51], it could be stated that the situation described in manufacturing processes could constitute an emerging risk. 

### 6.1. Major Accident and Occupational Accident

In regard to the definitions for ‘accident’ considered in this study, ‘major accident’ is defined by Directive Seveso III, while ‘occupational accident’ is not defined by the directives studied. Therefore, other sources are required. Besserman and Mentzer [12] consider that each country defines their statistics differently including different definitions for lost time incidents, non-fatal injuries, and what constitutes the manufacturing/chemical industry, among others. Furthermore, such authors indicate that there is minimal reporting of true process safety metrics resulting from the loss of containment of a hazardous substance. 

In any case, a major accident can also be considered to be an occupational accident if harm to workers exists. Inversely, an occupational accident can also be a major accident when it meets the requirements set out in Directive Seveso III. The causality relationship between both types of accident has not been studied here, although its interest in the sphere of safety is evident. In the 1970s, the effect of human actions and organizational factors on accident occurrence was recognized, but it took until the mid- 1980s before management became aware that they were key to achieving a good level of both occupational and process safety [52]. The incorporation of Bayesian networks into risk assessment may be another interesting focus for both research and industrial purposes, because it allows a systemic approach considering human error and management influences [49].

### 6.2. Links and Transitional Spaces

The main correspondence between risk management in both types of accidents is the presence in the manufacturing process of one or more of the hazardous substances included in Annex I of Directive Seveso III, for which directives derived from the Framework Directive are also applicable. The said directives may be understood as the deployment of this correspondence into further links that are more specific, giving rise to two types of transitional spaces: functional transitional spaces and regulatory transitional spaces.

Functional transitional spaces (that is, activities involving chemical agents, workplaces and installations) are interconnected as briefly outlined in Figure 3. Such interconnection must be understood as a basic outline that can be extended in different directions in the field of risk management as, for instance: the safety change management studied by Gerbec [53]; management of exposure to nanomaterials studied by Hunt et al. [54]; machine safety, which can be generally defined through Directive 2006/42/EC and the ISO 12100: 2010 and ISO/TR 14121-1: 2012 standards on safety of machinery [55,56] 

As regarding regulatory transitional spaces, they are defined by the intersections between the risks management systems studied through the Framework Directive, Directive Seveso III and the ISO 45001:2018 standard.

The links shown in Table 4 between management systems derived from the Framework Directive and Directive Seveso III, have been classified as: four are strong (Annex III sections (i), (ii), (iv) and (v)), two are weak (Annex III sections: ((iii) and (vi)), and one is non-existent (Annex III section (vii)).

This result is coherent under the perspective of the Framework Directive, which compiles a set of general guidelines that are further developed by specific directives, as shown in the results in Table 5. Therefore, the set established by the Framework Directive and individual directives completes and strengthens the seven links above, configuring the transitional space TS 1.

As regards the correspondence between management systems derived from the ISO 45001:2018 standard and Directive Seveso III, shown also in Table 4, 6 of these links have been classified as strong and one as relatively strong (sections Annex III: (iii)), configuring transitional space TS 2.

These results are also coherent with the aim of an OSH management system as defined by the ISO 45001:2018 standard. Moreover, these results make it possible to draw up a coherent correspondence between the structures of the ISO 45001:2018 standard [14] and the Framework Directive, configuring transitional space TS 3.

As regards TS 4, given that it may be considered to be a specific transitional space resulting from the intersection of three general transitional spaces, it is key to defining and channelling the transitions between systems by means of the corresponding individual directives in each case, including at least Directive 98/24/EC on chemical agents [7]. This consideration is still valid when the intersection occurs between two systems, and the linking and transitional role played by the individual directives is equally important.

The risk assessment techniques are a key structuring element between the functional and regulatory transition spaces, as shown in the results of Table 4 and, especially, of Table 5. However, according to Brocal et al. [9] it is necessary to deepen through future research on the analysis of differentiating and applicative criteria between the techniques used in the field of safety occupational and safety linked to major accidents.

These links and transitional spaces may facilitate system integration. In this regard, Li and Guldenmund [13] point out that according to the literature, an integrated management system is more advanced than independent safety systems, as safety is just one of the comprehensive organization management objectives.

In the process of integrating systems, besides the existence of transitional spaces, non-traditional spaces also exist as a result of the specific aspects of each system that have no direct correlation with the other systems. These non-transitional spaces have not been studied here, yet may be equally important in any integration process, since they define the frontiers necessary to avoid unwanted interference with the transitional spaces.

## 7. Conclusions

The main objective of this study has been met through identifying and analysing the links and transitional spaces between the risk management of occupational accidents and major accidents that involve hazardous substances in manufacturing processes. To this end, the risk management systems derived from Directive Seveso III, the Framework Directive and the ISO 45001:2018 standard [14] have been analysed, obtaining three main results. 

The first result of this analysis, the main link identified between the risk management of both types of accidents, is the presence in a manufacturing process of any of the hazardous substances included in Annex I of Directive Seveso III, for which the directives derived from the Framework Directive are also applicable, and the principles and guidelines of the ISO 45001:2018 standard are applicable on a voluntary basis [14].

As a second result, the intersection of Directive Seveso III, the Framework Directive and the ISO 45001:2018 standard, configures three general transitional spaces (TS 1, TS 2 and TS 3). 

In turn, and as a third result, the intersection of these three general transitional spaces configures a specific transitional space (TS 4), which is key to defining and channelling the transition between systems by means of the individual directives that may correspond in each case, which will, at least, include the directive on chemical substances. This will enable integration processes between the systems considered.

The above results are limited from a regulatory and technical perspective. In regard to the regulatory perspective, the context is limited to the EU, as well as Directive Seveso III, the Framework Directive and any individual directives that further develop it. Other directives and regulations that are especially relevant in the sphere of safety, as for instance the REACH Regulation [2] and CLP Regulation [3], are open to further analysis to enable their integration into the management systems. From a technical perspective, no distinction has been made between traditional risk management and emerging risk management. Similarly, no distinction has been made between static and dynamic approaches.

The results and limitations stated above may point towards future paths of research, from which models to integrate the risk management of occupational accidents and major accidents may be developed based on real experiences and data.

By way of final conclusion, the identification and analysis of the links and transitional spaces carried out by this study aspire to being a starting point that will inspire other researchers to continue and further develop this study with the end goal of efficiently integrating risk management systems related to accidents derived from dangerous substances in manufacturing processes. 

## Figures and Tables

**Table 1:** Individual directives within the meaning of Article 16 (1) of Directive 89/391/EEC on occupational safety and health (OSH).

Nr	Individual Directives	Topic	Year (First Publication)
1	Directive 89/654/EEC [15]	Workplace	1989
2	Directive 2009/104/EC [16]	Work equipment	1989
3	Directive 89/656/EEC [17]	Personal protective equipment	1989
4	Directive 90/269/EEC [18]	Manual handling of loads	1990
5	Directive 90/270/EEC [19]	Display screen equipment	1990
6	Directive 2004/37/EC [20]	Carcinogens or mutagens at work	1990
7	Directive 2000/54/EC [21]	Biological agents at work	1990
8	Directive 92/57/EEC [22]	Temporary or mobile construction sites	1992
9	Directive 92/58/EEC [23]	Safety and/or health signs	1992
10	Directive 92/85/EEC [24]	Pregnant workers	1992
11	Directive 92/91/EEC [25]	Mineral-extracting industries; drilling	1992
12	Directive 92/104/EEC [26]	Mineral-extracting industries	1992
13	Directive 93/103/EC [27]	Work on board fishing vessels	1993
14	Directive 98/24/EC [7]	Risks related to chemical agents at work	1998
15	Directive 99/92/EC [28]	Risks from explosive atmospheres	1999
16	Directive 2002/44/EC [29]	Vibration	2002
17	Directive 2003/10/EC [30]	Noise	2003
18	Directive 2004/40/EC [31]	Electromagnetic fields	2004
19	Directive 2006/25/EC [32]	Artificial optical radiation	2006
20	Directive 2013/35/EU [33]	Electromagnetic fields	2013

**Table 2:** Industrial safety technologies (IST) linked to individual directives within the meaning of Article 16 (1) of Directive 89/391/EEC (adapted from [35]).

CLASSIFICATION OF IST	DIRECTIVE 89/654/EEC WORKPLACE [15]	DIRECTIVE 2009/104/EC WORK EQUIPMENT [16]	DIRECTIVE 773/1997 PERSONAL PRO. EQUIPMENT [17]	DIRECTIVE 90/269/EEC MANUAL HANDLING LOADS [18]	DIRECTIVES 2004/37/EC AND 98/24/EC CHEMICAL [7]	DIRECTIVE 92/58/EEC SIGNAL. [9]	DIRECTIVE 99/92/EC EXPLOSIVE ATMOS. [28]
WORK EQUIPMENT	○	•	○	–	•	○	•
PLACES AND WORKPLACES	•	○	○	○	•	○	•
HANDLING, STORAGE AND TRANSPORT	•	•	○	•	•	○	•
ELECTRICITY	•	•	○	–	•	○	•
FIRES	•	•	○	–	•	○	•
CHEMICALS	•	•	○	○	•	○	•

• Direct link. ○ Cross link.

**Table 3:** Carcinogenic or mutagenic substances from Annex I, Part 2 of Directive Seveso III. Environmental value limits and related manufacturing processes.

DIRECTIVE SEVESO III: PART 2 [8]				CLP Regulation [36]		Examples of Manufacturing Processes [36]	VLA 2018 [37]	
Column 1	CAS number	Column 2	Column 3	Carcinogens or mutagens	Phrases H (Hazard)		VLA-ED ® (Reference value for Daily Exposure)	VLA-EC ® (Reference value for Short-term Exposure)
Dangerous substances		Qualifying quantity (tonnes) for the application of						
		Lower-tier requirements	Upper-tier requirements					
Arsenic pentoxide, arsenic (V) acid and/or salts	1303-28-2	1	2	Carc. 1A	H301,H331, H350, H410	Special glass manufacturing (light bulbs and tubes, optic glass, glass for liquid-crystal displays (LCD), etc.)	0.01 mg/m^3^	–
Arsenic trioxide, arsenious (III) acid and/or salts	1327-53-3	–	0,1	Carc. 1A	H300,H314, H350, H410	Manufacturing of low-melting glass and manufacturing of electronic components	0.01 mg/m^3^	–
Ethyleneimine	151-56-4	10	20	Carc. 1B, Muta. 1B	H225, H300, H310, H314, H330, H340, H350, H411	Manufacturing of ethyleneimine and aziridine polymers	0.2 ppm; 0.36 mg/m^3^	–
Formaldehyde (concentration ≥ 90%)	50-00-0	5	50	Carc. 1B	H301, H311,H314, H317, H331, H341, H350	Metal cutting, machining and grinding processes	0.3 ppm; 0.37 mg/m^3^	0.6 ppm; 0.74 mg/m^3^
Ethylene oxide	75-21-8	5	50	Carc. 1B, Muta. 1B	H220,H315,H319, H331, H340, H350	Glycol (ethylene glycol), polyglycol and polyol synthesis used in manufacturing fibres, coolants and foams	1 ppm; 1.8 mg/m^3^	–
Propylene oxide	75-56-9	5	50	Carc. 1B, Muta. 1B	H224, H302,H311, H319, H331, H335, H340, H350	Manufacturing of flexible or rigid polyurethane foams	2 ppm; 4.8 mg/m^3^	–
4, 4′-Methylene bis (2-chloraniline) and/or salts, in powder form	101-14-4	–	0,01	Carc. 1B	H302, H350, H410	Manufacturing of jet engine turbine blades, radar systems and home appliances	0.01 ppm; 0.1 mg/m^3^	–

**Table 4:** Correspondence between structures of risk management systems: Directive Seveso III, Framework Directive and ISO 45001:2018 Standard.

Directive Seveso III: Safety management system–Issues [8]		Framework Directive–Articles [6]	ISO 45001:2018 Standard–Sections [14]
(i) Organization and personnel	The roles and responsibilities of personnel involved in the management of major hazards at all levels in the organization, together with the measures taken to raise awareness of the need for continuous improvement.	Article 5. General provision Article 6. General obligations on employers Article 7. Protective and preventive services Article 11. Consultation and participation of workers Article 13. Workers’ obligations	Section 5.3 Organizational roles, responsibilities and authorities Section 10.3 Continual improvement
	The identification of training needs of such personnel and the provision of the training so identified.	Article 6 (6.3.d). General obligations on employers Article 12. Training of workers	Section 5.4 (e.4) Consultation and participation of workers
	The involvement of employees and of subcontracted personnel working in the establishment which are important from the point of view of safety.	Article 6 (6.4). General obligations on employers	Section 8.1.4.3 Outsourcing
(ii) Identification and evaluation of major hazards	Adoption and implementation of procedures for systematically identifying major hazards arising from normal and abnormal operation including subcontracted activities where applicable and the assessment of their likelihood and severity.	Article 6 (6.3.a). General obligations on employers Article 9 (9.1.a). Various obligations on employers	Section 6.1.2 Hazard identification and assessment of risks and opportunities
(iii) Operational control	Adoption and implementation of procedures and instructions for safe operation, including maintenance, of plant, processes and equipment, and for alarm management and temporary stoppages.	Article 6 (6.2). General obligations on employers Article 9 (9.1.b). Various obligations on employers Article 10. Worker information	Section 8.1.1 Operation planning and control Section 8.1.2 Eliminating hazards and reducing occupational safety and health (OSH) risks
	Taking into account available information on best practices for monitoring and control, with a view to reducing the risk of system failure.	Article 9 (9.1.c). Various obligations on employers Article 10. Worker information	
	Management and control of the risks associated with ageing equipment installed in the establishment and corrosion: inventory of the establishment’s equipment, strategy and methodology for monitoring and control of the condition of the equipment; appropriate follow-up actions and any necessary countermeasures.	–	–
(iv) Management of change	Adoption and implementation of procedures for planning modifications to, or the design of new installations, processes or storage facilities.	Article 6 (6.1 and 6.3.d). General obligations on employers	Section 8.1.3 Managing of change
(v) Planning for emergencies	Adoption and implementation of procedures to identify foreseeable emergencies by systematic analysis, to prepare, test and review emergency plans to respond to such emergencies and to provide specific training for the staff concerned. Such training shall be given to all personnel working in the establishment, including relevant subcontracted personnel.	Article 8. First aid, fire-fighting and evacuation of workers, serious and imminent danger	Section 8.2 Emergency preparedness and response
(vi) Monitoring performance	Adoption and implementation of procedures for the ongoing assessment of compliance with the objectives set by the operator’s major-accident prevention policy (MAPP) and safety management system, and the mechanisms for investigation and taking corrective action in case of non-compliance. The procedures shall cover the operator’s system for reporting major accidents or ‘near misses’, particularly those involving failure of protective measures, and their investigation and follow-up on the basis of lessons learnt. The procedures could also include performance indicators such as safety performance indicators (SPIs) and/or other relevant indicators.	Article 9 (9.1.c and 9.1.d). Various obligations on employers	Section 9.1 Monitoring, measurement, analysis and performance evaluation Section 10.2 Incident, nonconformity and corrective action
(vii) Audit and review	Adoption and implementation of procedures for periodic systematic assessment of the MAPP and the effectiveness and suitability of the safety management system.The documented review of performance of the policy and safety management system and its updating by senior management, including consideration and incorporation of necessary changes indicated by the audit and review.	–	Section 9.2 Internal audit

**Table 5:** Correspondence between structures of risk management systems of Directive Seveso III, Directive 98/24/CE on chemical agents and Directive 2004/37/EC on carcinogens or mutagens.

Directive Seveso III [8]	Directive 98/24/CE on chemical agents at work [7]	Directive 2004/37/EC on carcinogens or mutagens at work [20]
(i) Organization and personnel	Article 8. Information and training for workers	Article 11. Information and training of workers
(ii) Identification and evaluation of major hazards	Article 4. Determination and assessment of risk of hazardous chemical agents	Article 3. Scope–determination and assessment of risks
(iii) Operational control	Article 5. General principles for prevention of risks associated with hazardous chemical agents and application of this Directive in relation to assessment of risks Article 6. Specific protection and prevention measures	Article 4. Reduction and replacement Article 5. Prevention and reduction of exposure Article 8. Foreseeable exposure Article 9. Access to risk areas Article 10. Hygiene and individual protection
(iv) Management of change	Article 4. (4.2 and 4.5). Determination and assessment of risk of hazardous chemical agents Article 8. Information and training for workers	Article 3. Scope–determination and assessment of risks Article 11. Information and training of workers
(v) Planning for emergencies	Article 7. Arrangements to deal with accidents, incidents and emergencies	Article 5. Prevention and reduction of exposure Article 7. Unforeseen exposure
(vi) Monitoring performance		
(vii) Audit and review	Article 4. Determination and assessment of risk of hazardous chemical agents	Article 3. Scope–determination and assessment of risks
